# Does Differential Habitat Selection Facilitate Coexistence Between Badgers and Hedgehogs?

**DOI:** 10.1002/ece3.70744

**Published:** 2025-01-26

**Authors:** Katie A. Lee, Antonio Uzal, Louise K. Gentle, Philip J. Baker, Richard J. Delahay, Anthony Sévêque, Robert S. Davis, Richard W. Yarnell

**Affiliations:** ^1^ School of Animal, Rural and Environmental Science, Brackenhurst Campus Nottingham Trent University Southwell UK; ^2^ Health and Life Sciences Building, School of Biological Sciences University of Reading Berkshire UK; ^3^ National Wildlife Management Centre Animal and Plant Health Agency York UK; ^4^ Department of Wildlife, Fisheries and Aquaculture Mississippi State University Mississippi State Mississippi USA; ^5^ Department of Conservation Management Nelson Mandela University George Western Cape South Africa

**Keywords:** Intraguild predation (IGP), *mammals*, occupancy modelling, random encounter model, Spatiotemporal niche partitioning

## Abstract

Predicting the spatial and temporal responses of species exhibiting intraguild predation (IGP) relationships is difficult due to variation in potential interactions and environmental context. Eurasian badgers ( *Meles meles* ) are intraguild predators of European hedgehogs ( *Erinaceus europaeus* ) and are implicated in their population decline via both direct predation and competition for shared food resources. Previous studies have shown spatial separation between these species and attributed this to hedgehogs experiencing a ‘landscape of fear’, but little is known about the potential role of differential habitat use. We estimated the density and occupancy of both species at 22 rural study sites in England and Wales, to explore whether food availability, habitat or the presence of badgers, explained hedgehog distributions. Hedgehog density varied significantly across major rural land uses, whereas badger density did not. Although both species coexisted at a regional (1 km^2^) scale, occupancy modelling showed spatial segregation at a finer (individual camera trap) scale, associated with differential habitat use. In contrast to badgers, hedgehogs were recorded near buildings, and in areas supporting lower invertebrate biomass. This is in agreement with IGP theory, whereby IG‐prey may occupy suboptimal habitat to avoid predation; however, hedgehog habitat use did not vary relative to the presence of badgers. Badger and hedgehog temporal activity showed no evidence of separation. Although these findings are consistent with hedgehogs avoiding badgers via a landscape of fear, they are also indicative of differential habitat use, highlighting the need for more holistic studies considering variation in habitat selection and food availability when investigating intraguild relationships. Future studies exploring alternative hypotheses for urban habitat selection by hedgehogs are needed to better understand how possible spatial niche partitioning may support their coexistence with badgers in some areas.

## Graphical abstract

Eurasian badgers ( *Meles meles* ) are intraguild predators of European hedgehogs ( *Erinaceus europaeus* ) and are implicated in their population decline via both direct predation and competition for shared food resources. We estimated the density and occupancy of both species at 22 rural study sites in England and Wales, to explore whether food availability, habitat or the presence of badgers, explained hedgehog distributions. Hedgehog density varied significantly across major rural land uses, whereas badger density did not. Although both species coexisted at a regional (1 km^2^) scale, occupancy modelling showed spatial segregation at a finer (individual camera trap) scale, associated with differential habitat use. Badger and hedgehog temporal activity showed no evidence of separation. Although these findings are consistent with hedgehogs avoiding badgers via a landscape of fear, they are also indicative of differential habitat use, highlighting the need for more holistic studies considering variation in habitat selection and food availability when investigating intraguild relationships.

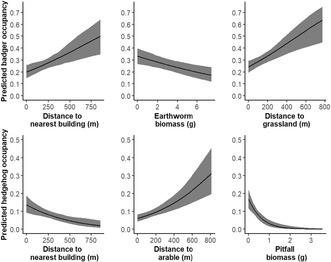

## 1 Introduction

Competition and predation occur simultaneously within an intraguild predation (IGP) relationship (Polis, Myers, and Holt 1989) and can lead to negative population responses in the prey species. Spatial, temporal and dietary partitioning provide mechanisms that can facilitate coexistence between intraguild (IG) competitors by reducing competition and/or the likelihood of predation (Sévêque et al. 2020). However, difficulty persists in disentangling species interactions caused by habitat preferences from those caused by IG‐competitors. Nevertheless, understanding what drives interactions amongst IG‐competitors, and their habitat preferences, is essential to ensure the impact of predation is not overstated and inappropriate conservation actions, such as predator control, are avoided.

IGP has been observed across all major animal groups including arthropods (Brown et al. 2015), birds (Sergio and Hiraldo 2008), fish (Bachiller et al. 2015) and mammals (Arim and Marquet 2004). In the simplest model of IGP, the IG‐predator gains a direct energetic advantage through consuming the IG‐prey, and by reducing competition for shared prey (Polis and Holt 1992). Consequently, the IG‐prey species may respond by occupying lower quality habitats to minimise the risk of predation (Morris 2009; Robinson, Bustos, and Roemer 2014).

IG‐competitors can coexist at different spatiotemporal scales (Pettett et al. 2018). For example, bobcats ( *Lynx rufus* ) may avoid coyotes ( *Canis latrans* ) when basal prey are abundant but are forced to use areas of higher coyote presence when prey resources in suboptimal habitats are diminished (Wilson et al. 2010). However, where coyotes are IG‐prey of grey wolves ( *Canis lupus* ), the former can occupy the same prey‐rich areas as wolves during winter by altering their daily activity, scavenging from wolf kills whilst temporally avoiding inter‐specific encounters (Arjo and Pletscher 1999). This demonstrates the interplay between different dimensions of niche partitioning that can impact the dynamics of IGP.

Variation in resource availability, and fluctuations in IG‐prey and/or predator densities, also leads to variation in prey responses to predation risk through different dimensions of niche partitioning. When food resources are limited, predation pressure on IG‐prey may increase (Polis and McCormick 1987); in response, prey spatially segregate themselves by occupying or preferentially utilising, suboptimal habitats (Lonsinger et al. 2017). Additionally, IG‐prey may consume alternative food resources, increasing the likelihood of coexistence through dietary partitioning (Balme et al. 2017). In areas where food resources are sufficiently abundant to support both predators and prey, temporal, rather than spatial, partitioning may provide an important mechanism for promoting coexistence (Pudyatmoko 2019).

Avoidance is a key mechanism facilitating the coexistence of some species, relying on the utilisation of either the same or alternative food resources, at different times or in different locations (Darmon et al. 2012). Hence, it is important that food availability and diet are considered in studies investigating mechanisms for coexistence within IGP relationships. However, studying these patterns requires multiple study sites, with a combination of habitats and varying food availability, to generate sufficient data to provide explanatory power (Scotson et al. 2017).

Eurasian badgers ( *Meles meles* ) and West European hedgehogs ( *Erinaceus europaeus* ) share an IGP relationship (Doncaster 1992), and increases in badger populations in England and Wales (Judge et al. 2017) have been linked to a recent decline in hedgehog populations (Hof, Allen, and Bright 2019). Both species compete for similar food resources (predominantly invertebrates), and badgers predate hedgehogs, thereby acting as the principal driver of interactions between the two species (Polis, Myers, and Holt 1989; Lee et al. In Prep). Badgers and hedgehogs are widely distributed across rural landscapes in Western Europe (Judge et al. 2017; Wembridge and Wilson 2018), with significant potential for co‐occurrence at a regional scale (Judge et al. 2017; Wembridge and Wilson 2018). However, at a finer scale, they exhibit different habitat associations, with hedgehogs showing an affinity for suburban areas not commonly shared by badgers (Young et al. 2006; Pettett et al. 2017). Such relationships have been attributed to badgers creating a ‘landscape of fear’ for hedgehogs, precluding them from using suitable habitat (Hof, Snellenberg, and Bright 2012). Indeed, hedgehog occupancy on a 1 km^2^ scale is negatively correlated with the presence of badger setts (burrows) in rural England and Wales (Yarnell et al. 2014; Williams et al. 2018), solidifying the consensus that badgers exert negative pressure on hedgehogs (Young et al. 2006; Trewby et al. 2014; Hof, Allen, and Bright 2019). However, whether this negative population response is a result of competition for food, direct predation or both, has not been determined. Furthermore, it is not known whether the observed use of suburban areas by hedgehogs arises because it is an optimal habitat, (Schmitz et al. 2017) or because it reflects avoidance of badgers in the surrounding habitat (Doncaster 1992). Similarly, whether hedgehogs can adjust their activity patterns to facilitate temporal partitioning in areas where they co‐occur with badgers is yet to be determined. Temporal partitioning would be expected where both species have similar spatially overlapping habitat use, with their shared prey (Schmitz et al. 2017).

The aim of this study was to better understand the intraguild relationship between badgers and hedgehogs and whether badgers are exerting an influence over hedgehogs distribution and abundance (Schmitz et al. 2017). Based on IGP theory (Polis, Myers, and Holt 1989) we expect to find support for one of the following three scenarios: (1) If two intraguild predators (badgers and hedgehogs here) share the same prey resource across the same landscape, we would expect spatial overlap to be high and associated with areas of high food resources and temporal partitioning to result. (2) If badgers are dominating hedgehogs through interference or exploitative competition, we would expect spatial partitioning where badgers occupy areas of high food resources, with hedgehogs occurring in areas of lower food resource, with temporal overlap remaining high. Finally, (3) if both badgers and hedgehogs have distinct habitat preferences, we would expect clear spatial separation of each species, high temporal overlap and for both species to occupy areas of high food resource.

## 2 Materials and Methods

### 2.1 Study Sites

**FIGURE 1:**
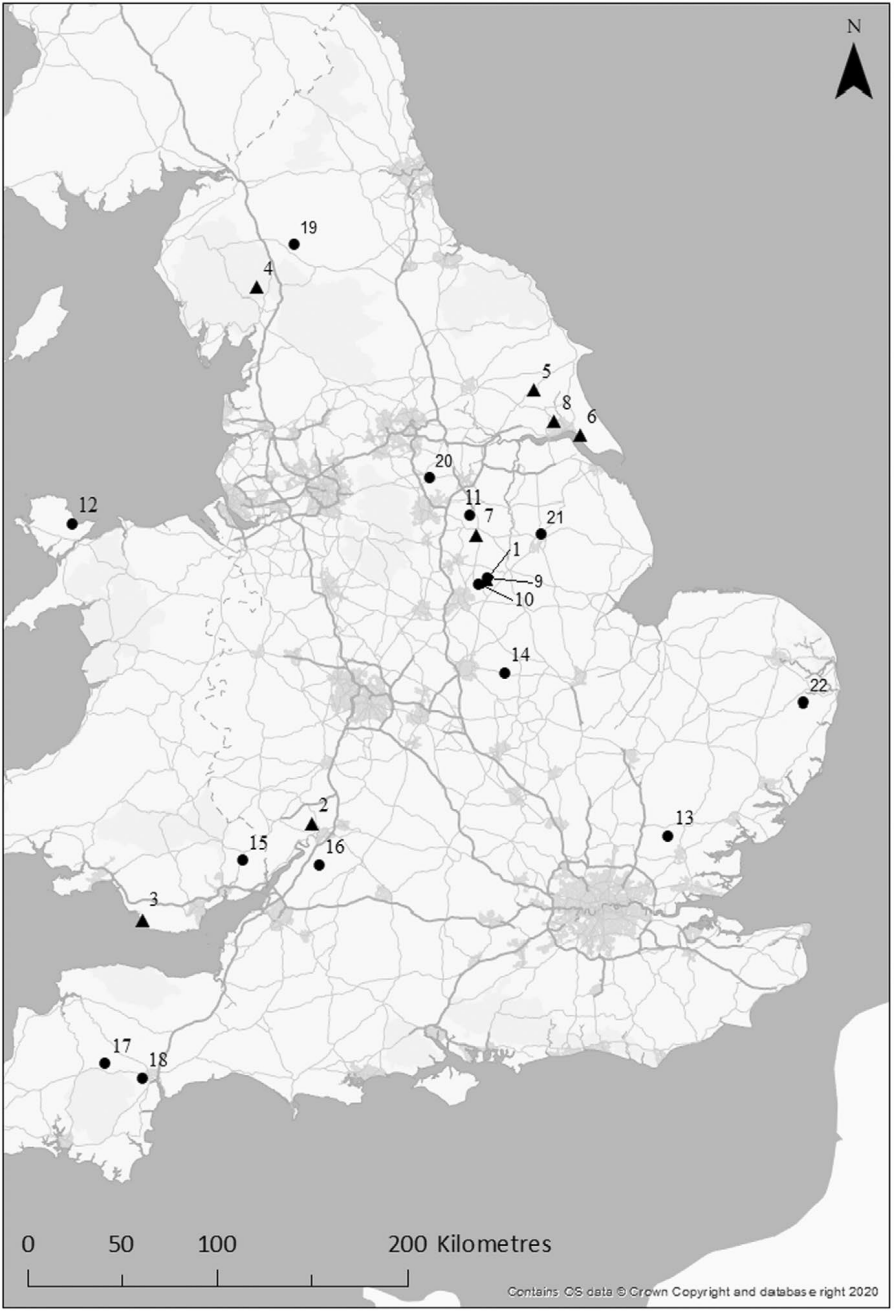
Location of 22 sites where camera and invertebrate surveys were conducted to investigate badger and hedgehog intraguild predation between April 2018 and September 2019. Sites surveyed in 2018 (*n* = 8) are depicted by triangles and those surveyed in 2019 (*n* = 14) by circles. Numbering depicts the order sites were visited.

We deployed 645 camera traps across 22 sites (mean = 29 per site), stratified by five key habitat categories: arable, amenity grassland (including residential gardens), agricultural grassland, built environment (buildings and hardstanding) and woodland (deciduous and coniferous). Twenty two rural sites (0.4–1.3 km^2^) were surveyed for badgers and hedgehogs between April and September in 2018 or 2019 (Figure 1). Camera trapping was used to estimate focal species occupancy and density, and invertebrate sampling was used to obtain an index of prey availability for both species. Rural study sites comprising either arable‐dominated, pasture‐dominated (where a single habitat represented ≥ 66.7% of the total area of a site) or mixed farming habitat were selected (Table 1), as these were considered landscapes most likely to be occupied by badgers and hedgehogs (Judge et al. 2014; Williams et al. 2018).

**TABLE 1:** Summary of site characteristics for 22 rural sites surveyed between April–September 2018–2019. Site numbers correlate with the sites illustrated in Figure 1. Invertebrate surveys and camera trapping surveys conducted for the first 10 nights of the initial survey period were used for occupancy analysis.

Site no.	Number of cameras deployed	Site name and county	Grid reference	Site area (km^2^)	Survey dates	No. of survey nights	Badger (Ü = present)	Hedgehog (ü = present)	Major land‐use description
1	40	Brackenhurst A—Nottinghamshire	SK 6952	0.5	05/04/2018–24/04/2018	19	Ü	ü	Mixed
2	38	Hartpury—Gloucestershire	SO 7822	0.8	30/04/2018–10/05/2018	10 or 11	Ü	ü	Mixed
3	40	Slade—Glamorgan	SS 8973	0.6	17/05/2018–28/05/2018	11	Ü	ü	Pasture
4	29	Kendal—Cumberland	NY 4903	0.4	18/06/2018–28/06/2018	10	Ü	ü	Pasture
5	29	Driffield—East Yorkshire	SE 9550	1.0	30/06/2018–10/07/2018	10	Ü	ü	Mixed
6	29	Keyingham—East Yorkshire	TA 2223	1.0	14/07/2018–24/07/2018	10	Ü		Mixed
7	20	Clumber—Nottinghamshire	SK 6275	0.7	03/08/2018–17/08/2018	14	Ü		Arable
8	27	Thorn—East Yorkshire	TA 1927	0.5	20/08/2018–30/08/2018	10	Ü		Arable
9	30	Brackenhurst B—Nottinghamshire	SK 6952	0.7	15/04/2019–26/04/2019	10	Ü	ü	Mixed
10	30	Epperstone—Nottinghamshire	SK 6649	0.8	07/05/2019–17/05/2019	10 or 11	Ü		Arable
11	25	Hodsock—Nottinghamshire	SK 6185	0.8	14/05/2019–24/05/2019	10	Ü	ü	Mixed
12	30	Anglesey	SH 5180	0.7	04/06/2019–14/06/2019	10		ü	Mixed
13	27	Dunmow—Essex	TL 6517	1.3	04/06/2019–14/06/2019	10 or11	Ü		Arable
14	30	Loddington—Leicestershire	SK 7902	1.3	17/06/2019–27/06/2019	10	Ü		Mixed
15	31	Usk—Monmouthshire	SO 4104	0.4	19/06/2019–29/06/2019	10 or 11	Ü	ü	Pasture
16	32	Woodchester—Gloucestershire	SO 8101	0.7	02/07/2019–12/07/2019	10	Ü		Mixed
17	25	Spreyton—Devon	SX 6997	0.6	16/07/2019–26/07/2019	10	Ü	ü	Pasture
18	24	Ide—Devon	SX 8990	0.5	17/07/2019–17/07/2019	10	Ü		Mixed
19	27	Knock—Cumberland	NY 6827	1.0	05/08/2019–15/08/2019	10	Ü		Pasture
20	26	Barnsley—South Yorkshire	SE 3905	1.0	09/08/2019–19/08/2019	10		ü	Mixed
21	30	Riseholme—Lincolnshire	SK 9678	0.9	12/09/2019–22/09/2019	10	Ü	ü	Mixed
22	26	Suffolk	TM 3687	1.2	16/09/2019–26/09/2019	10			Arable

### 2.2 Density Estimation and Occupancy Modelling

Sites were surveyed sequentially with 20–40 camera traps (Mean: 29), with 1‐2 sites studied at any one time. Collectively, 645 trap locations were utilised. Camera trap placement was stratified by five key habitats: arable, amenity grassland (including residential gardens), agricultural grassland, built environment (buildings and hardstanding) and woodland (deciduous and coniferous). The Sampling Tool in ArcGIS 10.6.1 (Environmental Systems Research Institute 2018) was used to generate random camera positions within each habitat. This meant cameras were deployed in proportion to the habitat availability in each study site.

Camera trapping surveys were conducted once per site, for a minimum of 10 consecutive 24‐h periods (Schaus et al. 2020). A handheld Garmin GPS 60 was used to locate randomly generated camera positions within 3–5 metres. However, practical constraints (e.g., impermeable fences, dense cereal crops or presence of livestock that would damage or interfere with camera operation) meant it was not possible to place all the cameras precisely at these random points, in which case the nearest suitable location within the same habitat was used.

Bushnell 119837 Essential E3 Trophy Cam HD (Bushnell Corp., Overland Park, KS, USA) cameras were used throughout the study to reduce any detection bias that may have arisen by using cameras with different technical specifications (Caravaggi et al. 2020). Cameras were attached to features (e.g., trees, hedgerows, telegraph poles, fence posts and wooden posts) that allowed a clear field of view in front of the camera, thereby providing a reasonable chance of detecting the target species (Rowcliffe et al. 2008); camera detection zones, specified by radius and angle, were calculated at individual camera locations (Cusack et al. 2015). Camera settings were as follows; mode = ‘Video’, LED = ‘High’, video size = ‘640 × 480 pixels’, video length = ‘15 s’, interval = ‘5 mins’, sensor level = ‘automatic’, night mode (dusk till dawn), time stamp = ‘On’, field scan = ‘Off’ and sound = ‘Off’. Each camera was fitted with a 16 GB micro‐SD card and placed in the ‘ON’ mode before positioning in the field. Cameras were placed approximately 30 cm above the ground. Cameras were checked after a minimum of 10 days and any that had failed during the survey period were removed from the study to avoid overestimating sampling effort. In total, eight hedgehog detections were removed across seven cameras that had retriggered within the 5‐min delay period, likely due to technical issues, or human error when setting up the cameras.

As invertebrates are known to constitute a major component of both hedgehog and badger diets (Hounsome and Delahay 2005; Lee et al. in Prep), an assessment of the macroinvertebrate community was used as an index of food availability. Invertebrate surveys were carried out at all sites, except for Thorn in 2018 (Site 8 in Figure 3) as particularly hot and dry conditions made digging pitfall traps challenging. Macroinvertebrate sampling effort was standardised across each site, comprising three earthworm cores and nine pitfall traps placed at random locations within each broad habitat type. Pitfall traps provided a standardised method for comparing ground dwelling macroinvertebrate communities (Boetzl et al. 2018), whilst soil cores provided an efficient means of assessing the relative abundance of earthworms with limited disturbance (Smith, Potts, and Eggleton 2008).

Plastic tapered cups (9.5 cm in height, 8 cm in width at the top and 6 cm at the base) were dug into the ground such that the rim of the cup was flush with the surface; this ensured a consistent likelihood of capture upon encounter with each cup, which was filled halfway with propylene glycol (Special Ingredients Ltd, Chesterfield, UK), an odourless preservative that is not harmful to livestock or other nontarget wildlife. The preservative also prevented predation amongst captured macroinvertebrates (Schmidt et al. 2006). A wooden stick was placed in each cup to provide a ramp for nontarget species such as small mammals and amphibians to escape. Each trap was covered by a 15 by 15 cm plastic half pipe, with two sections removed to leave a support in each corner, to prevent the traps flooding and to minimise disturbance. Traps were left unattended for 10 consecutive 24‐h periods before being collected and the contents stored until identification took place. Individual pitfall traps were sorted through and invertebrates > 5 mm long were identified to Order level and counted. Organisms from individual pitfall traps were dried in an oven at 60°C for 72 h and total dry mass for each Order was recorded.

Soil cores were taken following the protocol described by Valckx et al. (2011), and each 25 cm^3^ square soil core was thoroughly sorted by hand in the field. Earthworm abundance (number of earthworms counted) and wet biomass (g) were recorded before returning organisms to the environment. Invertebrate data were pooled within each habitat at each site for analyses.

The density of badgers and hedgehogs at each site was estimated from camera trap data using the Random Encounter Model (REM) (Rowcliffe et al. 2008). REM allows density estimates to be obtained for species that do not have unique identifying features (Schaus et al. 2020). Density (D) is calculated as a function of trap rate (the number of detections (*y*) per unit time (*t*): *y*/*t*), speed of movement (*v*), radial distance to the animal (*r*) and camera detection zone (*θ*):

$$D=\frac{y}{t}\;\frac{\pi }{\mathrm{vr}\left(2+\theta \right)}$$

Total trapping effort was calculated by multiplying the number of trapping hours per survey night, defined as the time between the first and last detection, from all detections of each target species at each site, by the number of survey nights. A 5‐min delay between camera triggers was considered sufficient to assume that video recordings represented unique detection events. A minimum of 10 independent detections per species was required to calculate reliable density estimates using site‐specific parameters (Rowcliffe et al. 2008). The position of the animal at the first point of detection was used to calculate distance from the camera and angle of detection. Landmark features such as trees, shrubs and hedgerows were important for estimating the position of the animal and the path it travelled within the video footage. The movement trajectory of the animal was measured using a measuring tape and the overall distance travelled (m) was recorded. Speed of movement (m/s) for each species was calculated as mean across all detections at each site. For sites where a species was detected but the number of detections was low (*n* < 10), mean parameters for species' daily ranges were calculated from data across all sites. This meant 4 out of 12 hedgehog densities and 5 out of 19 badger densities were derived from using average parameters.

The 95% confidence intervals around the density estimates were calculated by resampling camera locations within each site using replacement bootstrapping analysis based on 1000 iterations (Rowcliffe et al. 2008). Standard errors were also calculated for the independent estimation of speed of movement (*v*), radial distance to the animal (*r*) and camera detection zone (*θ*). Due to the variability in hedgehog densities, both linear and nonlinear approaches were used to investigate the relationship with badger density. A linear regression was used to investigate whether hedgehog density was associated with badger density. The scatterplot of standardised predicted values versus standardised residuals indicated that the data met the assumptions of homogeneity of variance and linearity, and the residuals were approximately normally distributed. However, a nonlinear Gamma GLM of hedgehog and badger density failed to converge, likely due to the small number of sites where the species co‐occurred (Montez‐Rath et al. 2006).

Inferential analyses were performed to assess whether major land‐use at each site affected badger and/or hedgehog density. Major landuse at each site was classified as either being Arable, Mixed or Pasture (Table 1). Assumptions of normality were not met, so the nonparametric Kruskal–Wallis test (Kruskal and Wallis 1952) was used to compare hedgehog and badger density between different land‐use types. Pairwise comparisons of densities of each species in each land‐use type were assessed using Bonferroni‐adjusted Wilcoxon rank sums tests. A one‐way ANOVA (Chambers, Freeny, and Heiberger 1992) was performed to assess whether mean badger density varied with land‐use as the assumptions of normality were met.

Occupancy modelling estimates the probability of a species being present whilst accounting for imperfect detection (MacKenzie et al. 2017). Repeated surveys allow the detection probability to be estimated, either as a constant or using detection covariates. For occupancy analysis, each camera trap location was defined as a site, thereby allowing potential spatial segregation at the habitat scale to be assessed, and each survey night was treated as a repeat survey. Where possible, cameras were spaced a minimum of 40 m apart, such that individuals could have visited > 1 camera location per night, potentially violating assumptions of independence. However, occupancy was calculated as a measure of relative activity at a site rather than of true occupancy (MacKenzie et al. 2017). Detection for badgers and hedgehogs was treated as constant, as there was no evidence to show it would vary with any of the covariates. Camera locations across all study sites were pooled prior to analysis, to provide single occupancy estimates for both badgers and hedgehogs.

Data for occupancy modelling were collated from 618 of the 645 cameras deployed, as 27 cameras placed at the Thorn site were discounted because of missing invertebrate data. All occupancy analyses were conducted in the R Statistics software (R Development Core Team 2019) using the package ‘unmarked’ (Fiske et al. 2013).

To assess the occurrence of each species in relation to habitat availability (amenity grassland, arable, buildings (within built environment), agricultural grassland and woodland: Table 2), nearest Euclidean distances (m) from each camera location to each habitat type were included as covariates in occupancy models. Distances were calculated in ArcMap 10.6.1 using the ‘Near’ tool. Distance to amenity grassland and distance to the nearest building were co‐linear, therefore only distance to buildings was included in the occupancy models, so as to reflect the level of urbanisation across each rural site. Covariates consisting of continuous data were standardised using *z*‐scores (Table 2).

**TABLE 2:** Summary of the covariates used in the single‐season single‐species occupancy models and the data format for each.

Variable name	Description	Variable type
Dist_to_arable	Distance from camera location to nearest habitat feature—Arable	*Z*‐scores
Dist_to_building	Distance from camera location to nearest habitat feature—Buildings	*Z*‐scores
Dist_to_grassland	Distance from camera location to nearest habitat feature—Grassland	*Z*‐scores
Dist_to_woodland	Distance from camera location to nearest habitat feature—Woodland	*Z*‐scores
Earthworm_biomass	Camera location specific estimate of earthworm biomass	*Z*‐scores
Pitfall_biomass	Camera location specific estimate of pitfall biomass	*Z*‐scores

To test whether the availability of food was associated with the occurrence of either species, the abundance and biomass of both earthworms and pitfall trap captures within each habitat was used as an index of prey availability; mean values for each measure were calculated for each habitat type on each study site (*n* = 21). Also, we calculated values of food availability for each camera location (*n* = 618) by extracting the proportion of each broad habitat within a 10 m radius (maximum distance either species was detected from a camera) and weighting the average abundance and biomass of earthworms and pitfall trap captures by these proportions. This resulted in four different measures of food availability per camera location; earthworm wet biomass, earthworm abundance, pitfall abundance and pitfall capture dry biomass.

Because badgers and hedgehogs rarely co‐occurred in any study sites, penalised multispecies occupancy models were used to investigate how environmental factors influenced their distribution and to assess the influence of interspecific interactions on occupancy (Clipp et al. 2021). Penalised occupancy models add a penalty (λ) to the likelihood value to shrink estimates of parameters and associated standard errors towards zero and correct unreasonably large parameter estimates. Following Clipp et al. (2021), cross‐validation scores (crv) were used to identify the value of λ that resulted in the best model fit. The possible values for λ varied from 0 (i.e., unpenalised likelihood) to the following discrete values: {0.01, 0.02, 0.04, 0.08, 0.16, 0.32, 0.64, 1.28, 2.56, 5.12}. The value of λ that resulted in the highest cross‐validation score (i.e., the best predictive score) was used to fit the penalised likelihood to the multispecies occupancy models.

To determine correlates of occupancy for each species, a full model was initially constructed to test distance to arable, distance to building, distance to grassland, distance to woodland, earthworm biomass and pitfall biomass. Interaction was defined as null (~0) as the initial model focussed on the occupancy of each species separately and did not test for interactions between them. This model produced a value for detection probability, naïve occupancy (occupancy rate without correcting for imperfect detection) and estimated occupancy (considering the detection probability).

Further multispecies models were then used to evaluate the effect of interactions between hedgehogs and badgers. These models included the best occupancy parameters yielded for each species from the full occupancy model (i.e., only covariates for which the 95% confidence interval did not cross zero). We compared models with no interaction, constant interaction and those for which interaction depended on shared influential covariates (i.e., covariates that had an effect on the occupancy of both species). The top model was deemed to be the one with the best cross‐validation score (Clipp et al. 2021).

### 2.3 Temporal Activity Analysis

We used the time and date stamp from camera trap detections to describe diel activity patterns (percentage of time spent active over a 24‐h period) and temporal overlap of hedgehogs and badgers across sites. As day length can impact species activity levels, we accounted for this by using average anchoring to transform clock time to solar time using the ‘solartime’ function in the package ‘activity’ (Vazquez et al. 2019). To minimise bias due to small sample sizes, a minimum of 10 detections per species at each site were required to fit kernel density estimates and calculate the coefficient of overlap (Lashley et al. 2018). Analyses were conducted using the packages ‘activity’ (Rowcliffe 2019) and ‘overlap’ (Meredith and Ridout 2016) in R.

We estimated the coefficient of overlap (Δ) between hedgehogs and badgers at sites where both species were detected and the number of observations were ≥ 10 for each species (*n* = 4 sites). High confidence in activity estimation requires 100 detections per species (Rowcliffe et al. 2014; Dykes et al. 2018), but lower levels of detection in the present study meant that site‐level analyses could not be performed. We tested whether the presence of badgers altered hedgehog temporal behaviour by estimating the temporal overlap between hedgehogs at sites where badgers were present (*n* = 4) and absent (*n* = 2). The ‘overlap’ package produces a nonparametric estimator of Δ, ranging from 0 (no overlap) to 1 (complete overlap). We considered a threshold of Δ > 0.75 to be representative of high temporal overlap (Monterroso, Alves, and Ferreras 2014). We estimated confidence intervals for each Δ via smoothed bootstrapping with 10,000 resamples (Meredith and Ridout 2016). Following Ridout and Linkie (2009), we used ∆_1_ for pairwise comparisons where either sample size was < 75 detections and ∆_4_ when sample sizes were ≥ 75 detections. We tested for differences in pairwise comparisons of daily activity patterns using a Wald test performed using the ‘compareAct’ function in package ‘activity’ (Ridout and Linkie 2009).

## 3 Results

Hedgehogs were detected at 12 sites (Table 3), 10 of which also contained badgers. Badgers were found at 19 sites, of which 9 did not contain hedgehogs. All 10 sites where both species were found consisted of either mixed livestock and arable farms or livestock farms. One site (Site 12: Suffolk) did not contain either species; this was the largest arable‐dominated site in the study.

**TABLE 3:** Camera trap derived density estimates for badgers and hedgehogs across 22 sites in England and Wales between April 2018 and August 2019. Site number, number of camera detections and the estimated Day Range used to calculate random encounter model (REM) density estimates are also given. Activity represents the proportion of time animals spent being active and is one of the parameters required to obtain REM estimates. Standard deviations and 95% confidence intervals are given for density estimates.

Site	Land‐use	Hedgehog					Badger				
		*N*	DR	D	SD	95% CI	*N*	DR	D	SD	95% CI
1	Mixed	11	0.46	8.21	3.55	3.41–17.23	53	1.23	6.62	2.12	3.38–11.5
2	Mixed	40	0.71	16.5	4.62	8.52–27.48	16	1.1	5.36	1.43	2.83–8.52
3	Pasture	16	0.6	7.16	2.33	4.05–12.88	26	2.21	2.58	0.93	0.75–4.42
4	Pasture	2	0.52	0.85	1.22	0.44–3.53	42	0.74	30.29	8.1	16.3–48.09
5	Mixed	9	0.52	5.41	0.74	4.12–7.05	36	0.75	12.8	4.09	6.44–23.09
6	Arable	0		0			62	0.47	57.83	13.12	35.86–91.92
7	Mixed	0		0			22	1.07968	7.14	2.36	3.94–13.01
8	Arable	0		0			3	0.81	0.89	0.1	0.72–1.16
9	Mixed	14	1.53	2.12	1.02	0.84–4.43	33	0.5	18.32	9.36	4.98–39.89
10	Arable	0		0			8	0.81	1.39	0.52	0.76–2.64
11	Mixed	31	0.85	11.38	4.2	4.93–21.48	1	0.81	0.35	0	0.35–0.35
12	Mixed	12	0.67	8.12	0.43	7.36–9.6	0		0		
13	Arable	0		0			50	0.95	18.51	4.75	11.16–30.21
14	Mixed	0		0			22	0.86	16.96	17.02	3.99–64.07
15	Pasture	10	0.56	6.65	2.19	2.27–8.09	1	0.81	0.56	0	0.56–0.56
16	Mixed	0		0			35	1.15	7.33	1.66	4.89–11.44
17	Mixed	1	0.52	0.27	0	0.27–0.27	18	1.33	5.61	1.83	3.01–10.25
18	Pasture	0		0			14	1.95	2.95	0.71	1.77–4.86
19	Pasture	0		0			23	0.57	12.59	3.01	8.06–18.92
20	Mixed	16	0.45	13.89	3.48	8.27–22.05	0		0		
21	Mixed	6	0.52	4.29	0.77	3.11–5.8	7	0.81	2.36	0.64	1.3–3.49
22	Arable	0		0			0		0		

Of the 618 unique camera locations included in the occupancy analysis across 21 study sites, 225 were operational in 2018 and 393 in 2019. There was evidence of a significant association between landscape type and hedgehog (χ^2^_2_ = 8.09 *p* = 0.02) but not badger (χ^2^_2_ = 1.06, *p* > 0.05) presence. Hedgehogs were not detected at the five sites classified as arable‐dominated, but were detected in 60.0% of the five pasture sites, and 75.0% of the 12 mixed farm sites. Badgers were present at 80.0% of the five arable sites, 100.0% of the five pasture sites and 83.3% of the 12 mixed farm sites.

### 3.1 Numerical Relationship Between Badgers and Hedgehogs

**FIGURE 2:**
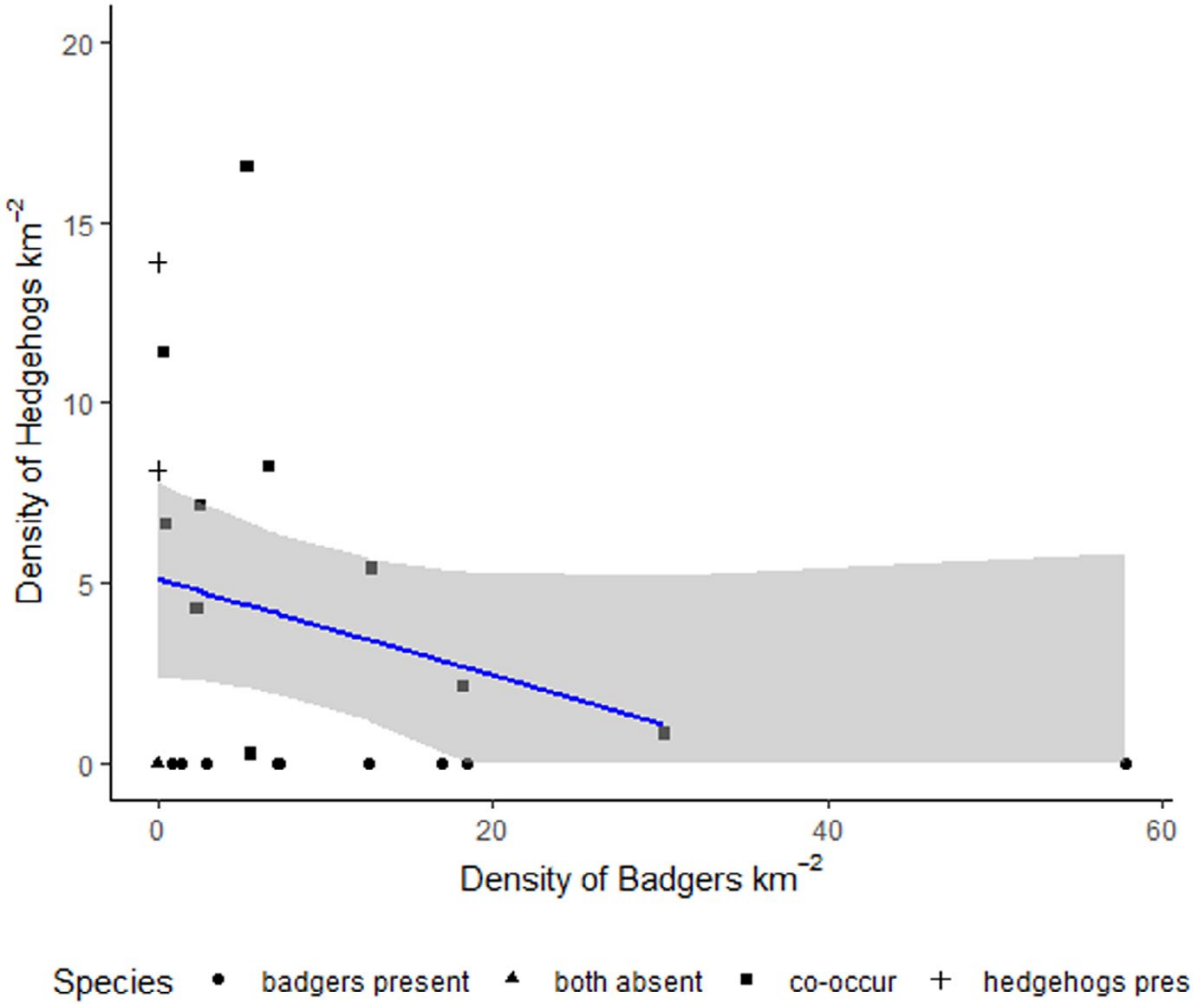
Scatterplot showing the relationship between badger and hedgehog density (km^−2^) across 22 rural sites in England and Wales. The blue line shows the regression line of density estimates, with 95% bootstrapped confidence intervals represented by grey shading.

Density estimates were derived for hedgehogs and badgers at 12 and 19 sites, respectively (Table 3). Site‐specific parameters were incorporated into density estimates at sites where detections exceeded 10 per species and average activity parameters (derived from all sites) were used for sites with low detection levels (< 10 detections per species). Hedgehog and badger densities ranged from 0.3 to 16.5 km^‐2^ and from 0.4 to 57.8 km^‐2^, respectively. Hedgehogs were absent from a site where badger density exceeded 30.3 individuals km^‐2^. Hedgehogs were not detected at 10 sites and badgers were not detected at three sites, suggesting they were either absent or below the limit of detection (Dorazio et al. 2006); where either species was not detected at a site, its density was assumed to be zero. A linear regression was used to investigate whether hedgehog density was associated with badger density, which showed a nonsignificant negative relationship (*R* ^2^ = 0.08, *F*_1,20_ = 2.71, *p* = 0.12). However, bootstrapped confidence intervals did not include zero, suggesting a relationship between badger and hedgehog densities could be statistically significant (*β* = –0.132, 95% CI [–0.34, –0.03]) (Figure 2).

### 3.2 Land‐Use and Habitat Associations

**FIGURE 3:**
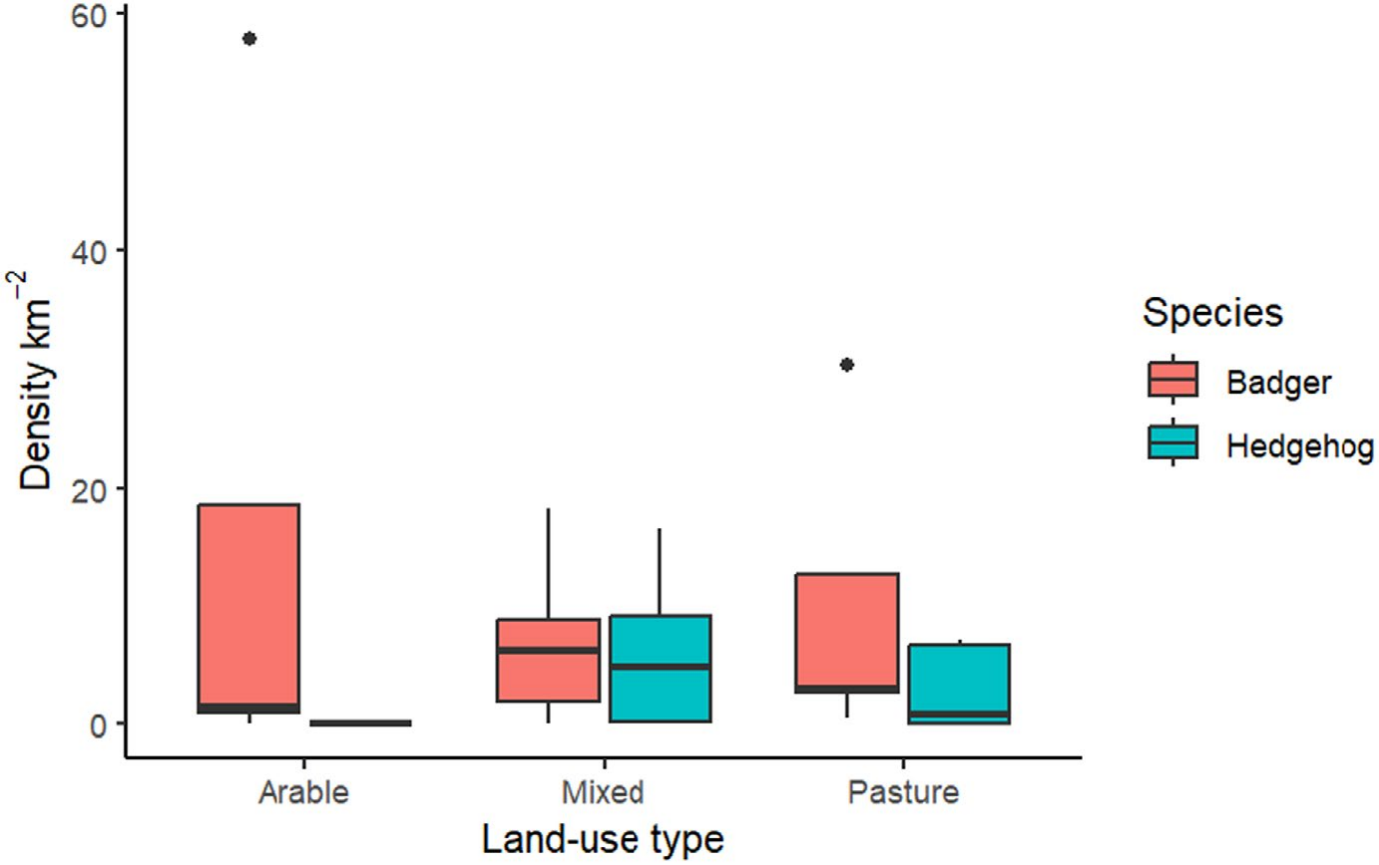
Boxplots (median, 25% and 75% quartiles, and 95% confidence interval) of the density of hedgehogs (blue) and badgers (pink) in Arable (*n* = 5), Mixed farming (*n* = 12) and Pasture (*n* = 5) dominated landscapes.

Hedgehog density was significantly higher in mixed farmland landscapes than in arable‐dominated landscapes (*post hoc*, *p* = 0.04), adjusted using Bonferroni correction (Figure 3). In contrast, there was no significant difference in mean badger density, between the three land‐use types (ANOVA) (*p* > 0.05).

### 3.3 Factors Predicting Hedgehog Occupancy

**FIGURE 4:**
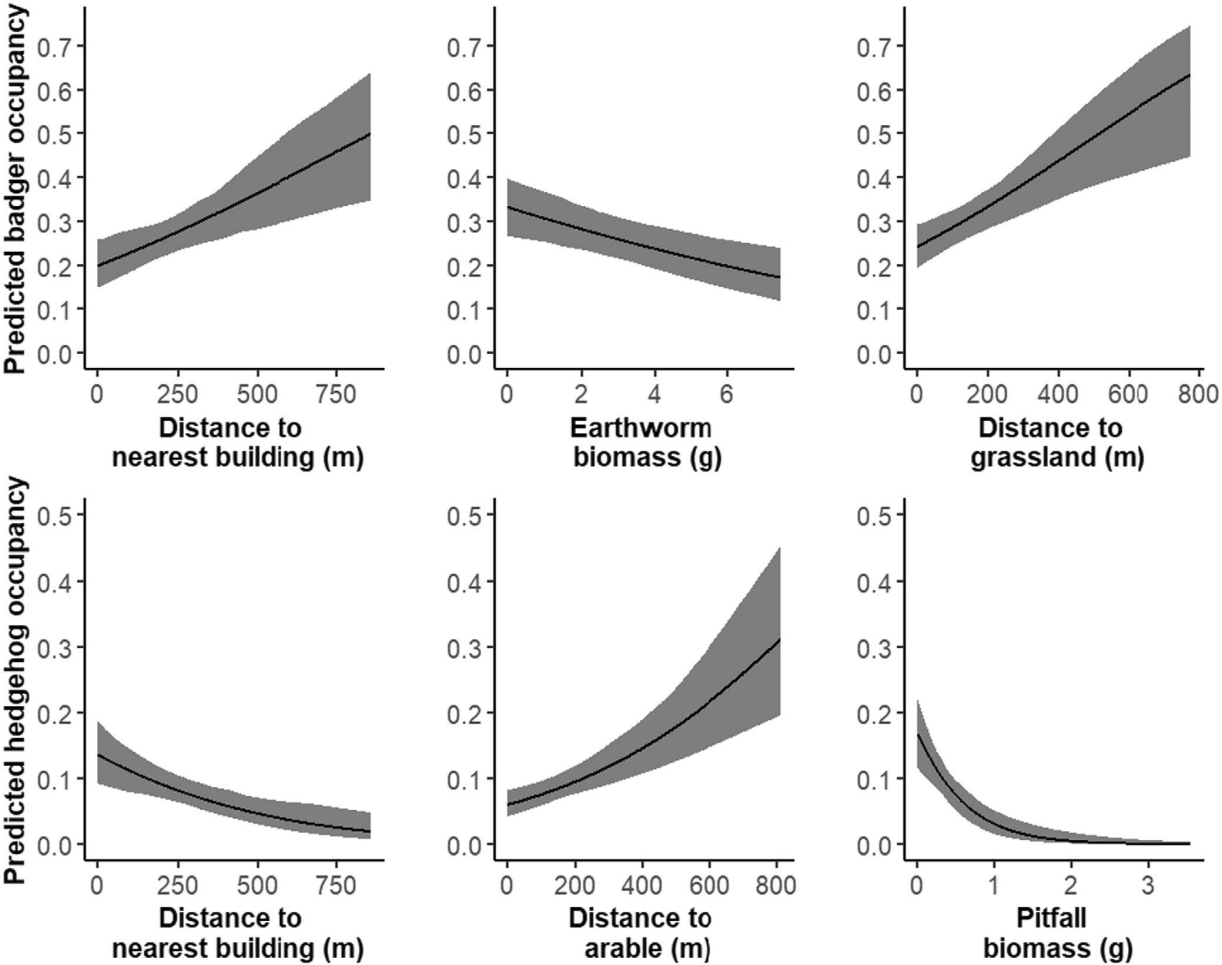
Relationship between significant environmental covariates from Occupancy modelling for badger and hedgehog occupancy across 618 camera location in England and Wales in 2018–2019. Predicted badger occupancy relationships are on the top row, whilst hedgehog relationships are on the bottom row. The significant environmental covariates for badgers were from left to right, distance to nearest building, earthworm biomass and distance to grassland. Significant environmental covariates for hedgehogs were from left to right, distance to buildings, distance to arable fields and pitfall biomass.

Naïve hedgehog occupancy across the 618 camera locations was 10.0%. Of the 62 camera locations where hedgehogs were detected, 40 (64.5%) were positioned < 200 m from buildings. Hedgehog occupancy was higher as distance to buildings decreased (*β* = –0.32, 95% CI [–0.57, –0.07]) and distance to arable habitat increased (*β* = 0.31, 95% CI [0.08, 0.54]). Hedgehog occupancy was negatively associated with pitfall capture biomass (*β* = –0.66, 95% CI [–0.92, –0.40]) (Figure 4, Table 4) but was unrelated to earthworm biomass.

**TABLE 4:** Summary of full single‐species model using penalised likelihood for badgers and hedgehogs, with best likelihood penalty λ = 2.56. Covariates for which 95% confidence intervals did not cross zero are shown in bold and were retained to build the multi‐species model.

	Variable	Estimate	95% CI	*Z* value	*p*
**Occupancy**					
Badger	(Intercept)	−0.87	[−1.07, −0.68]	−8.648	0.000
	**dist_building**	**0.29**	**[0.12, 0.47]**	**3.255**	**0.001**
	dist_arable	−0.13	[−0.34, 0.08]	−1.223	0.221
	dist_woodland	−0.08	[−0.28, 0.11]	−0.830	0.406
	**dist_grassland**	**0.27**	**[0.09, 0.45]**	**2.872**	**0.004**
	**worm_biomass**	**−0.20**	**[−0.38, −0.03]**	**−2.258**	**0.024**
	pitfall_biomass	−0.03	[−0.19, 0.14]	−0.331	0.741
Hedgehog	(Intercept)	−2.08	[−2.34, −1.83]	−16.019	0.000
	**dist_building**	**−0.32**	**[−0.57, −0.07]**	**−2.553**	**0.011**
	**dist_arable**	**0.31**	**[0.08, 0.54]**	**2.696**	**0.007**
	dist_woodland	0.01	[−0.23, 0.24]	0.054	0.957
	dist_grassland	−0.02	[−0.26, 0.22]	−0.174	0.862
	worm_biomass	0.24	[0.00, 0.48]	1.956	0.050
	**pitfall_biomass**	**−0.66**	**[−0.92, −0.40]**	**−4.903**	**0.000**
Detection					
Badger	(Intercept)	−1.43	[−1.22, −1.64]	−13.35	0.000
Hedgehog	(Intercept)	−1.50	[−1.81, −1.19]	−9.59	0.000

### 3.4 Factors Predicting Badger Occupancy

Badgers were detected at 162 (26.2%) of the 618 camera locations. Of these, 81% were within 200 m of arable habitat, whilst only 33% were located within 200 m of buildings. Badger occupancy was higher further from buildings (*β* = 0.29, 95% CI [0.12, 0.47]) and distance to grassland (*β* = 0.27, 95% CI [0.09, 0.45]). Badger occupancy was negatively associated with earthworm biomass (*β* = –0.20, 95% CI [–0.38, 0.03]) (Figure 4, Table 4) but unrelated to pitfall biomass.

### 3.5 Multispecies Occupancy

Badgers were recorded at more individual camera trap locations than hedgehogs (*n* = 162 (25.0%) and *n* = 67 (10.4%), respectively). At the 11 sites where badgers and hedgehogs were detected, only 1.7% of camera trap locations (*n* = 11) recorded both species, suggesting fine‐scale spatial separation within sites. Distance to building was the only covariate influencing both hedgehog and badger occupancy (see above). The best ranking multispecies model showed constant interaction between the occupancy of badgers and hedgehogs (crv = –1674.6), followed by an interaction dependent on distance to building (crv = –1674.9) and no interaction (crv = –1676.2). The best predictive score for all three models was obtained with λ = 2.56.

### 3.6 Temporal Analyses

**FIGURE 5:**
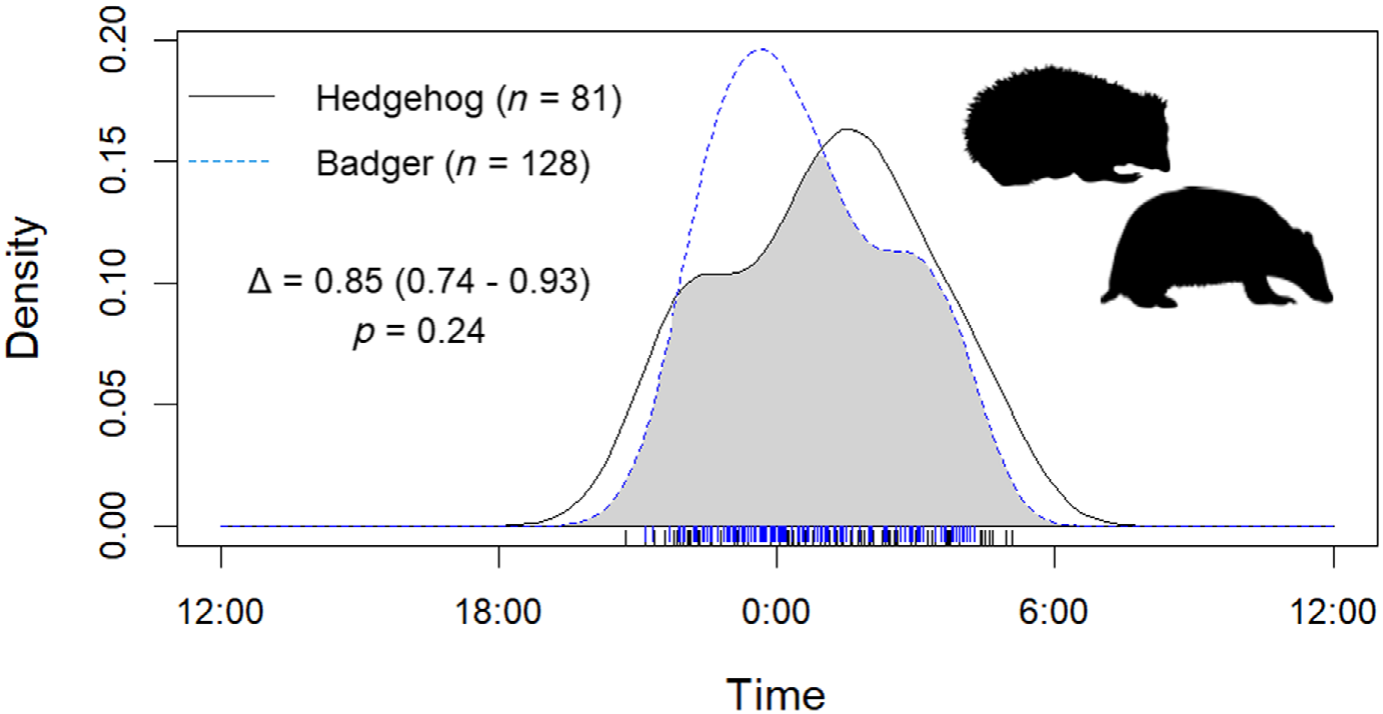
Overlap plot comparing diel activity pattern density curves of badger and hedgehog as obtained from camera trap detections (81 hedgehog and 128 badger detections) across four sites where they co‐occured in England and Wales. The grey shaded area indicates activity overlap and individual detection times at shown along the *X* axis as rings.

**FIGURE 6:**
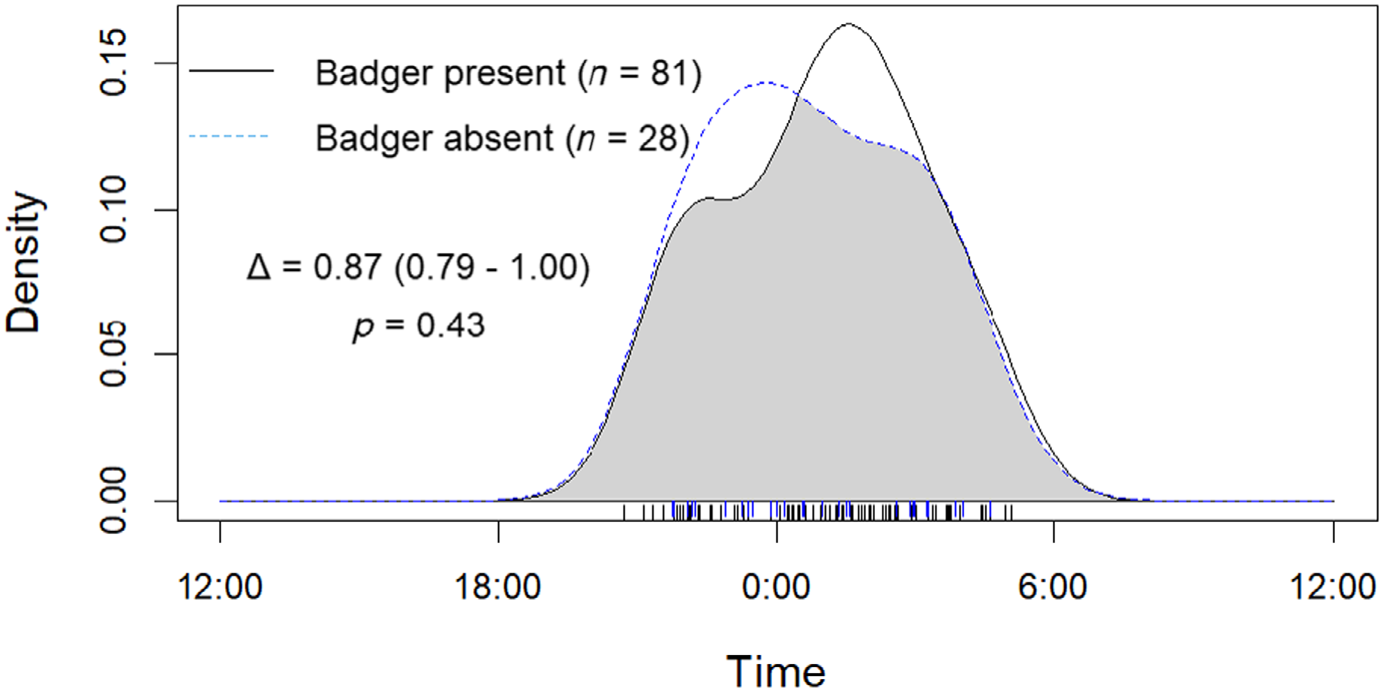
Overlap plot comparing diel activity pattern density curves of hedgehog detections at camera locations where badgers were present (*n* = 81) and locations where badgers were absent (*n* = 28). The grey shaded area indicates activity overlap and individual detection times at shown along the *X* axis by rings.

Overall activity levels were similar for hedgehogs (0.28 ± 0.02, 95% CI [0.23, 0.32]) and badgers (0.28 ± 0.01, 95% CI [0.25, 0.30]). Of the 168 hedgehog detections, 140 (83.3%) were on sites where badgers were also detected and 28 (16.7%) were on sites where badgers were not recorded. At sites where both species were present and there were sufficient detections, there was a high level of temporal overlap (Δ_4_ = 0.85, 95% CI [0.74, 0.93]: Figure 5) and no significant difference in activity levels (Wald *P* > 0.05). Hedgehog temporal activity at sites with and without badgers exhibited a high degree of overlap (Δ_1_ = 0.87, 95% CI [0.79, 1.00]: Figure 6) and no significant difference in activity levels (Wald *P* > 0.05).

## 4 Discussion

The results of this study demonstrate the importance of incorporating measures of both prey and habitat availability when studying population density and spatiotemporal relationships of intraguild predators and prey. Our results indicated that, at a site level, hedgehog density was significantly higher in mixed farmland than in arable landscapes (Wembridge and Langton 2015), that the spatial distribution of badgers and hedgehogs was associated with differences in habitat use and prey availability resulting in spatial separation, which negated the need for temporal avoidance, but that hedgehogs were absent (or below a minimum detectable density) where badger density exceeded 30.3 individuals km^‐2^.

Occupancy modelling based on detections at individual camera locations demonstrated that hedgehog presence and badger presence were predicted best by models that included measures of habitat association in combination with indices of food availability. The penalised likelihood multispecies models did not detect any strong signal of species interaction, and instead their respective distribution and spatial segregation seemed to be dictated by differential habitat use. Hedgehogs were associated with prey‐poor habitats (as measured by the biomass of terrestrial invertebrates in pitfall traps) and proximity to buildings, whereas badgers were more likely to occur at greater distances from buildings and grassland, but closer to arable and woodland habitats. Such spatial segregation may negate the necessity for temporal avoidance and was consistent with the high overlap in periods of activity observed for the two species. These patterns are consistent with badgers precluding hedgehogs, pushing them into suboptimal habitats (Young et al. 2006), but also support an alternative hypothesis of species‐specific habitat preferences.

Previous comparisons of indices of hedgehog abundance and badger sett density suggested that hedgehogs would be largely absent from rural areas with high sett densities (Micol, Doncaster, and Mackinlay 1994; Young et al. 2006; Williams et al. 2018). However, using sett density as a proxy for badger abundance is likely to be less reliable than using directly comparable estimates of badger and hedgehog density, because of the wide variation in social group size across different landscapes (2.67–7.92 individuals: Judge et al. 2017). The current study is, therefore, the first to directly quantify the numerical relationship between badgers and hedgehogs using locally derived density estimates for both species. In this study, the maximum density of badgers where hedgehogs were also recorded was 30.3 km^‐2^ (Site 4), but they were absent at the only other site where badgers were recorded at a higher density (57.8 km^‐2^; Site 6).

Therefore, depending on badger group sizes, these estimates may align with the suggestions of Williams et al. (2018) that hedgehogs may currently be excluded from areas where sett density exceeds 5.21 setts km ^−2^ and suggest that badger density, rather than badger presence alone, may influence the likelihood of coexistence with hedgehogs. Nevertheless, the badger density threshold for coexistence may be site‐specific, based on the availability of habitat, abundance of local food resources, and may depend on the scale at which coexistence is being assessed (Yarnell and Pettett 2020).

In the present study, hedgehog densities were significantly higher on mixed, rather than arable dominated, farmland sites, suggesting that the extent of arable landuse is likely an important determinant of hedgehog distribution and abundance across rural England and Wales. These results support the findings of Williams et al. (2018) that suggest large areas of the rural landscape may be largely uninhabitable for hedgehogs due to increased agricultural intensification. The extensive use of insecticides, coupled with the removal of hedgerows and grassy field margins, has led to an increasingly homogenised and fragmented rural landscape, which is perceived to have led to a decline in the availability of habitats which offer an abundance of food and resting sites for hedgehogs, as well as refugia from predators (Williams et al. 2018).

Hedgehogs have previously been shown to be associated with buildings, with higher densities found in urban than in rural areas (Hubert et al. 2011; Schaus et al. 2020), and an affinity for residential areas even in rural environments (Pettett et al. 2017). Residential areas increase habitat complexity, which may decrease the strength of interactions between IG‐competitors (Janssen et al. 2007) by creating more opportunities for differential habitat selection, which in this instance might result in a lower encounter rate between badgers and hedgehogs (Goldberg et al. 2022). Also, the positive association between hedgehogs and buildings may reflect greater resource availability (Pettett et al. 2017) including anthropogenic food resources (Yarnell et al. 2014; Pettett et al. 2017; Yarnell and Pettett 2020) in residential areas. Further quantitative studies on the relative abundance of natural prey and the extent of artificial supplementary feeding in rural and urban habitats are needed to better understand how variation in food resources may influence habitat selection by hedgehogs.

Whilst other studies have shown trends regarding the affinity between hedgehogs and ‘green infrastructure’ associated with the built environment (Hof, Snellenberg, and Bright 2012; Yarnell et al. 2014; Williams et al. 2018; Schaus et al. 2020), the question remains as to whether this is driven by a landscape of fear, with hedgehogs seeking refuge in habitat where badgers are likely to be less commonly encountered, or because it offers the best quality habitat in terms of shelter and food availability. If hedgehogs were primarily choosing their foraging habitat based on the avoidance of badgers, we might expect their habitat preferences to differ in relation to badger presence, such that they might occupy sub‐optimal habitat supporting poorer prey availability where encounters with badgers were more likely. In the present study, hedgehog occupancy was negatively correlated with pitfall trap invertebrate biomass. Moreover, arable habitat supported the highest abundance of beetles, as measured by pitfall trapping, and yet hedgehog occupancy decreased with proximity to arable habitat, indicating avoidance. However, as hedgehogs were only present at two sites where badgers were absent, further assessment of sites unoccupied by badgers is needed to ascertain whether patterns of hedgehog habitat selection are influenced by the presence of badgers. Nonetheless, no hedgehogs were found in arable‐dominated study sites, suggesting that this land use type is unsuitable for hedgehogs in England and Wales.

Arable‐dominated landscapes are not a preferred habitat of badgers (Hofer 1988); however, badger occupancy was associated with shorter distances to arable and woodland habitat and, in contrast to hedgehogs, with greater distances from buildings, possibly reflecting different foraging strategies of the two species (Huck, Davison, and Roper 2006; Pettett et al. 2017). Also, badger occupancy was associated with areas supporting lower earthworm biomass, despite earthworms being their principal prey (Macdonald et al. 2004), suggesting that other factors may have caused badgers to avoid earthworm rich habitat, such as grassland habitats. Badgers are generally more common in rural than urban areas (Harris 1984) but sett density in one urban area (Davison et al. 2009) was shown to be comparable with that in rural areas (Davison et al. 2008). However, the avoidance of amenity grassland and built‐up habitat in the present study, suggests the distribution of badger setts is not ubiquitous across rural and urban landscapes, perhaps indicating a greater wariness of proximity to humans amongst rural badgers.

This is the first study to investigate temporal partitioning in badger and hedgehog activity patterns. Previous studies have shown negative short‐term physiological responses of hedgehogs to badger odour (Ward et al. 1996; Ward, MacDonald, and Doncaster 1997) and alteration of their movements in the presence of badgers (Hof, Snellenberg, and Bright 2012), suggesting that hedgehogs might avoid times of high badger activity. However, our results indicated a high degree of temporal overlap in nocturnal activity of the two species.

The absence of temporal avoidance may suggest that spatial partitioning is sufficient to reduce competitive and predatory pressure on hedgehogs. Consistent with this, the present study demonstrated a clear difference in habitat use by the two species that could reflect segregation at a fine spatial scale (Hubert et al. 2011; Pettett et al. 2017), providing an additional hypothesis to the landscape of fear caused by badgers. For example, badgers avoided buildings, possibly due to fear of humans (Sévêque et al. 2020), thus creating pockets of low predation risk in habitats that are suitable for hedgehogs, and where supplementary food may be provided by humans (Hubert et al. 2011). However, hedgehogs were consistently associated with proximity to buildings in the present study regardless of whether badgers were present or not. This suggests that these areas are not simply a refuge from badgers, but likely provide resources such as greater food availability that are important to hedgehogs (Schaus et al. 2020).

Future studies should aim to confirm or refute the landscape of fear hypothesis by assessing hedgehog habitat use in a broader range of sites unoccupied by badgers, as only two sites where badgers were absent but hedgehogs were present were available in the present study. Moreover, to further understand the dynamic balance between predation and competition between badgers and hedgehogs, it would be useful to quantify the effects of food availability on predation rates. This was beyond the scope of the present study and would require long term monitoring of hedgehog and badger abundance and movement, with contemporaneous measures of food availability.

In summary, this study sought to investigate the association between badgers and hedgehogs to better understand factors associated with their coexistence in some areas, despite observed hedgehog declines in the UK rural environment. Our study showed a weak negative relationship between badger and hedgehog density and spatial separation between the species at a local scale. These findings are consistent with previous research suggesting that hedgehogs select habitats based on a landscape of fear created by badgers. However, we also showed that hedgehog and badger occupancy patterns were strongly related to the availability of different habitats and not natural food resources, potentially confounding conclusions about the drivers of hedgehog habitat selection. Data on activity levels suggest that spatial segregation may provide the mechanism for reducing competitive and predatory risk, negating the need for temporal avoidance. Furthermore, these results are consistent with low hedgehog occupancy and density in the rural landscape across England and Wales, likely due to a combination of factors rather than an increase in badgers per se. Importantly, we document situations where both species can co‐occur in mixed farming and pastoral landscapes at a 1 km^2^ scale, though arable landscapes appear unsuitable for hedgehogs regardless of badger presence. More broadly, this study highlights the need to include habitat and other resources, such as food, in studies investigating IGP to ensure that the roles of predation and competition are not overemphasised.

## Author Contributions

**Katie A. Lee:** conceptualization (equal), data curation (lead), formal analysis (equal), investigation (equal), methodology (equal), project administration (lead), writing – original draft (equal), writing – review and editing (equal). **Antonio Uzal:** conceptualization (equal), formal analysis (equal), writing – original draft (equal), writing – review and editing (equal). **Louise K. Gentle:** conceptualization (equal), formal analysis (equal), writing – original draft (equal), writing – review and editing (equal). **Philip J. Baker:** conceptualization (equal), writing – review and editing (equal). **Richard J. Delahay:** conceptualization (equal), writing – review and editing (equal). **Anthony Sévêque:** formal analysis (equal), methodology (equal), writing – review and editing (equal). **Robert S. Davis:** formal analysis (equal), methodology (equal), writing – review and editing (equal). **Richard W. Yarnell:** conceptualization (equal), formal analysis (equal), funding acquisition (lead), writing – original draft (equal), writing – review and editing (equal).

## Conflicts of Interest

The authors declare no conflicts of interest.

## Data Availability

The data that support the findings of this study are openly available in Dryad at: https://doi.org/10.5061/dryad.sn02v6xfn.
